# Retraction: Allopregnanolone Modulates GABAAR-Dependent CaMKIIδ3 and BDNF to Protect SH-SY5Y Cells Against 6-OHDA-Induced Damage

**DOI:** 10.3389/fncel.2022.882351

**Published:** 2022-03-18

**Authors:** Frontiers Editorial Office

The journal retracts the 13 January 2020 article cited above.

Following publication, concerns were raised regarding the integrity of the data in the published figures. The authors failed to provide a satisfactory explanation during the investigation, which was conducted in accordance with Frontiers' policies.

This retraction was approved by the Chief Editors of Frontiers in Cellular Neuroscience and the Chief Executive Editor of Frontiers. The authors agree to this retraction.

